# Correlational Analysis of ALS Progression and Serum NfL Measured by Simoa Assay in Chinese Patients

**DOI:** 10.3389/fneur.2020.579094

**Published:** 2020-12-03

**Authors:** Kazuo Sugimoto, Yi Han, Yuebo Song, Ying Gao

**Affiliations:** ^1^Department of Neurology, Dongzhimen Hospital, Beijing University of Chinese Medicine, Beijing, China; ^2^Institute for Brain Disorders, Beijing University of Chinese Medicine, Beijing, China

**Keywords:** amyotrophic lateral sclerosis, neurofilament light chain, motor neuron degeneration, clinical relevance, biomarker, single molecule array

## Abstract

**Background:** Neurofilament light chain (NFL) was believed to be a promising biomarker for the diagnosis of Amyotrophic lateral sclerosis (ALS) and disease burden evaluation.

**Objective:** To determine the serum NFL level and its clinical relevance, including its association with disease severity [evaluated by the ALS Functional Rating Scale–revised (ALSFRS-r) score and King's College staging system] and progression (evaluated by the disease progression rate (DPR) and diagnostic delay), in ALS patients in China.

**Method:** Serum NFL levels were detected using the Single Molecule Array (Simoa) technology in 30 ALS patients and 20 healthy controls (HCs).

**Results:** There were significantly elevated levels of serum NFL in patients with ALS than in the HCs *(P* < 0.001). The serum NFL levels were significantly higher in rapidly progressive ALS and patients in Stage 3 than in slowly progressive ALS and patients in Stage 2 (*P*_DPR_ < 0.001, *P*_Diagnosticdelay_ = 0.019; *P*_*stage*_= 0.033). Furthermore, the serum NFL levels negatively correlated with the diagnostic delay (*R*^2^ = 0.23, *P* = 0.016), the ALSFRS-r score (*R*^2^ = 0.15, *P* = 0.047) and disease duration (*R*^2^ = 0.15, *P* = 0.034), and positively correlated with the DPR (*R*^2^ = 0.42, *P* < 0.001).

**Conclusions:** The present study preliminarily investigated the diagnostic value of serum NFL and its clinical relevance in the Chinese ALS population using the ultrasensitive Simoa technology. The results demonstrated that the level of serum NFL may become a potential biomarker for ALS diagnosis and indicate disease severity and progression.

## Introduction

Amyotrophic lateral sclerosis (ALS) is a rare, disabling, and fatal neurodegenerative disease involving upper motor neurons that comprise the corticospinal tract and lower motor neurons that originate in the brain stem nuclei and ventral roots of the spinal cord (1–3). The disease generally has an insidious onset and a progressive disease course (4, 5). To date, the diagnosis of ALS has primarily been based on clinical symptoms and electrodiagnostic findings, and this process is very challenging in the early stages of the disease (1). As a result, a final correct diagnosis is typically delayed by at least 14 months from symptom onset, which leads to missing the optimal treatment time and inappropriate treatments during the period when the disease is undiagnosed (1, 4, 6, 7). In addition, after the diagnosis of ALS has been made, an efficient evaluation of the neurodegenerative burden, such as the disease severity and progression, are crucial for an appropriate medical plan that would be beneficial for improving the patient's quality of life and prolonging their survival. Currently, there are several clinical parameters that are applied to assess the disease severity and progression of ALS, among which the ALS Functional Rating Scale-revised (ALSFRS-r) score reflects the neurological disability. In addition, the King's College staging system evaluates the anatomical extension of the disease. These have both been widely used in clinical practice for disease severity appraisals, and the disease progression rate (DPR) and diagnostic delay have been used as disease progression indicators (8–10).

Of note, valid biofluid markers have not yet been applied in clinical practice, but they are urgently needed for diagnosis, disease severity, and progression evaluations. Currently, among the numerous candidate biomarkers for ALS, the neurofilament light chain (NFL) is believed to be the most promising alternative (11, 12). NFL is a cytoskeletal protein of large-diameter axons (>5 μm) of neurons (13). An elevated concentration of NFL in the cerebrospinal fluid (CSF) or blood could reflect axonal degeneration and neuron injury, and therefore indicate the presence of ALS (14). However, due to the comparatively low sensitivity of the traditional enzyme-linked immunosorbent assay (ELISA) and low concentration of NFL in the biofluid, especially in the blood, NFL assessments and their clinical application as a biomarker for ALS have been hindered in the past (15). With the development of a novel detection technique for NFL, a recent assay on the Single Molecule Array (Simoa) platform that possesses a valid high sensitivity has now become a reliable measure for NFL in biofluid (9, 15, 16). In particular, blood-derived NFL has proven to be an easily accessible biomarker with prognostic value for ALS (11, 17). In addition, an increasing amount of evidence has demonstrated that the NFL levels in the serum of ALS patients increases significantly compared to healthy or disease controls (11, 14, 17). Moreover, several studies that have focused on the clinical relevance of NFL in serum have reported that the serum NFL is closely correlated with disease severity and progression (10, 11, 14, 18). Of note, genetic and environmental factors have also been proven to play a vital role in the clinical characteristics, prognosis, and application of the biochemical marker in ALS (19, 20). Nevertheless, to our knowledge, there has still been no investigation of the serum NFL level and its clinical relevance in Chinese ALS patients that has used the Simoa technology, particularly that links the serum NFL level with the disease stage. Therefore, in this study, the Simoa technology is used to: determine the serum NFL levels in Chinese ALS patients and healthy controls (HCs); and to examine the clinical relevance of the serum NFL in Chinese ALS patients involving the association between the serum NFL level and indicators of disease severity (evaluated using the ALSFRS-r score and disease stage) and progression (evaluated using DPR and diagnostic delay).

## Methods

### Patients and Clinical Information

A total of 30 patients with ALS and 20 HCs were enrolled at the Department of Neurology, Dongzhimen Affiliated Hospital, Beijing University of Chinese Medicine, Beijing, China from 2018 to 2020. Patient consent was obtained for all participants. This study was approved by the research ethics committee of the Dongzhimen Affiliated Hospital, Beijing University of Chinese Medicine. Patients with ALS had a diagnosis of definite ALS according to the revised El Escorial criteria (21). Demographic, clinical, and laboratory data were collected retrospectively. A complete history, such as the date of disease onset, the date of ALS diagnosis and the sites of onset, and the ALSFRS-r score, disease stage, and forced vital capacity (FVC) at sampling, were reviewed. The disease severity was evaluated using the ALSFRS-r score (16) and the King's College staging system, which were both assessed by expert neurologists blinded to any biochemical results (8, 22, 23). According to the King's College staging system, ALS staging was based on the presence of symptoms such as wasting, weakness, spasticity, dysphagia, or dysarthria in different regions of the central nervous system (CNS), defined as the bulbar, upper limb, lower limb, or diaphragmatic (8, 24). Involvement in one of the above CNS regions was defined as Stage 1. Functional involvement of a second region or a third region was defined as Stage 2 or Stage 3. Swallowing impairment that involved gastrostomy and respiratory dysfunction that required non-invasive ventilation was Stage 4. Stage 5 was death (8). The King's College staging system defined Stage 2A as an ALS diagnosis and Stage 2B as the functional involvement of a second region. However, Stages 2A and 2B may have occurred concurrently when the diagnosis was made. Therefore, Stages 2A and 2B were merged into Stage 2, and the diagnosis of ALS was defined. Disease progression was evaluated using the DPR [(48–ALSFRS-r score at the time of sampling)/months elapsed between disease onset and sampling] and the diagnostic delay (the time interval from symptom onset to diagnosis) (10). The disease duration of ALS patients was defined as the time between the first symptom and blood sampling. Based on the site of symptom onset, patients were classified as having either bulbar onset (the onset of symptoms was in the bulbar region) or spinal onset (the onset of symptoms was in the cervical, thoracic, or lumbar regions). Additionally, based on median values of the DPR and diagnostic delay, the patients were dichotomized into “rapidly progressive” ALS and “slowly progressive” ALS.

### Sample Collection and Analysis

Blood samples were collected in tubes using a coagulating agent within 6 h of patient admittance. The samples were then centrifuged for serum at 3,000 r/min for 15 min within 2 h of blood sampling, and the serum was immediately frozen and stored at −80°C until assayed. The serum NFL was quantified using an ultra-sensitive Simoa technology (Quanterix, MA, US) on the automated Simoa HD-X platform (GBIO, Hangzhou, China) according to the manufacturer's instruction. The human NF-light assay (Cat No: 102258) kits were purchased from Quanterix and used accordingly. Serum samples were diluted at a 1:4 ratio for this assay. Calibrators and quality controls were measured in duplicate. All sample measurements were performed on a single run basis. The operators were unaware of the participants' disease status and clinical information.

### Statistical Analysis

A non-parametric test with the Steel-Dwass multiple comparison was used to compare three or more groups, and the Mann–Whitney U test was used for comparison between two groups. A Pearson's correlation was used for the correlation analysis between the NFL serum level and clinical parameters (despite disease stage). A Spearman's rank correlation coefficient was used for the correlation analysis between the disease stage and other clinical parameters, since the disease stage is an ordered categorical variable. The receiver operating characteristic (ROC) curves were used to discriminate between the ALS group and the HCs and examine the predictive discriminating values. The Youden index (sensitivity + specificity – 1) was calculated to determine the cut-off value, which maximizes the discrimination accuracy. Furthermore, to evaluate and adjust for the influence of patient demographic features, such as age and sex, a univariate analysis with a general linear model was employed, setting the demographic features as the covariate. A Fisher's exact test was used to test all categorical data. *P* < 0.05 were considered as statistically significant. The statistical analysis was performed using SPSS 21.0 Software, JMP13.0 software, and GraphPad Prism7 software.

## Results

### Clinical Characteristics of the ALS Patients

The demographic and clinical characteristics of the enrolled subjects are listed in Table 1. There were 21 males (70.0%) and nine females (30.0%) in the ALS group, and five males (25.0%) and 15 females (75.0%) in the HCs. There was a significant difference in age at sampling (*P* < 0.001) and sex (*P* = 0.003) between the ALS group and the HCs. Among the ALS patients, the median ALSFRS-r was 33.0, the median DPR was 0.49/month, and there were 10 patients (33.3%) in disease Stage 2, 11 patients (36.7%) in disease Stage 3, and nine patients (30.0%) in disease Stage 4 at sampling. In addition, 22 patients (73.3%) had spinal onset and 20 patients (66.7%) were being treated with Riluzole at the time of serum collection.

**Table 1:** Clinical information of patients with ALS and HCs.

	**ALS**	**HCs**
	***n* = 30**	***n* = 20**
Male, *n* (%)	21 (70.0)	5 (25.0)
Age^*^	59.0 (52.5–64.0)	26.0 (24.0–45.8)
Disease duration (months)^*^	17.8 (12.4–29.1)	NA
Diagnostic delay (months)^*^ (*n* = 25)	8.0 (4.9–12.0)	NA
Site of onset		NA
Spinal onset, *n* (%)	22 (73.3)	
Bulbar onset, *n* (%)	8 (26.7)	
ALSFRS-r^*^ (*n* = 26)	33.0 (29.3–42.0)	NA
DPR^*^ (*n* = 26)	0.49 (0.25–0.83)	NA
FVC^*^ (*n* = 18)	74.1 (70.0–79.0)	NA
Clinical stage, *n* (%)		NA
Stage 2	10 (33.3)	
Stage 3	11 (36.7)	
Stage 4	9 (30)	
Riluzole, *n* (%)	20 (66.7)	NA

*ALS, amyotrophic lateral sclerosis; ALSFRS-r, amyotrophic lateral sclerosis functional rating scale revised; DPR, Disease progression rate; FVC, forced vital capacity; HCs, Healthy controls; NA, not available; NFL, Neurofilament light chain*.

* *Data are presented as median (interquartile range)*.

### Serum NFL Levels in Patients With ALS and the HCs

**Figure 1:**
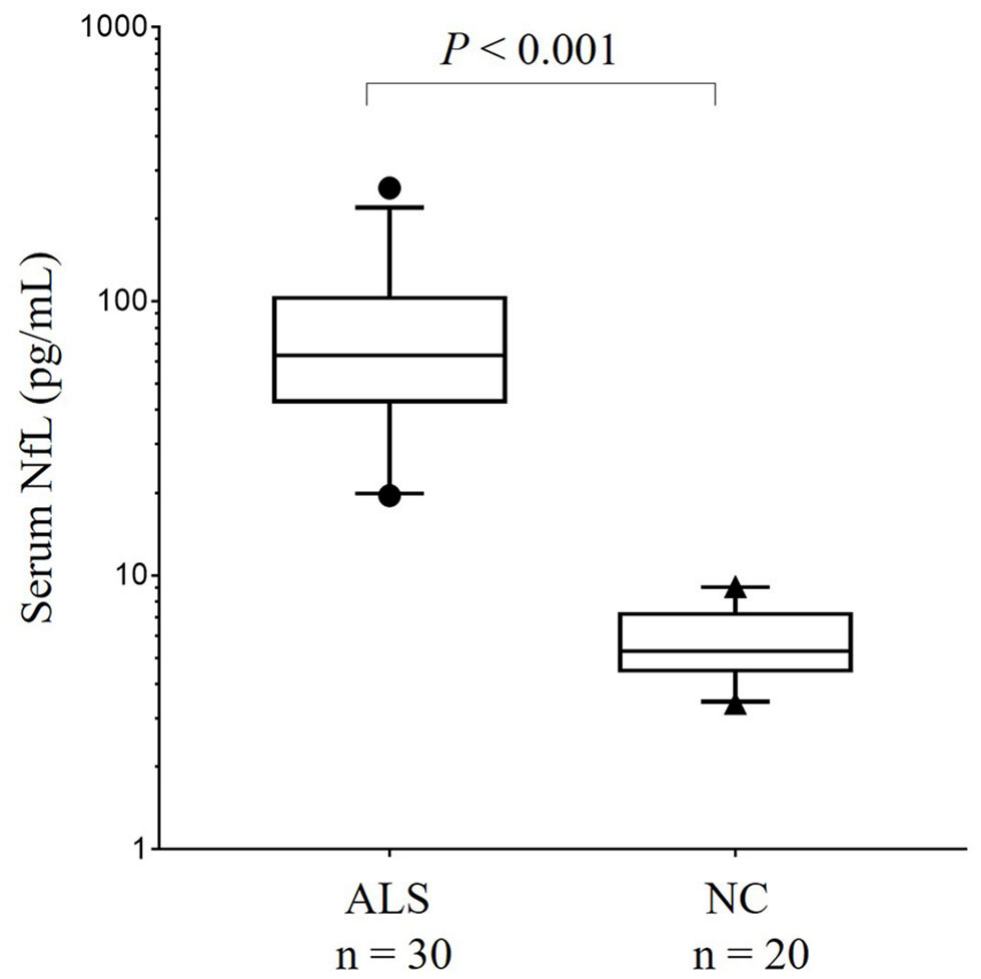
The comparison of serum NFL concentration between patients with ALS and HCs in China. Serum NFL concentration in patients with ALS and HCs. Given are the median concentrations, 25th and 75th percentiles, and 5 and 95% whiskers. Points represent values below 5% percentile and above 95% percentile. ALS, Amyotrophic lateral sclerosis; HCs, Healthy controls; NFL, Neurofilament light chain.

Due to the significant differences in age at sampling and sexes between the ALS group and the HCs, the influence of age at sampling and sex on the comparison of the serum NFL levels between the groups was evaluated using a general linear model. The results showed that both sex (*P* = 0.008) and age (*P* = 0.011) had significant influences in the comparison of NFL serum levels between the ALS patients and the HCs. After adjusting for the factors of age and sex, the results still showed a significant difference in NFL serum concentrations between the ALS patients and the HCs {median [interquartile range (IQR)]: 63.3 (46.9–98.1) pg/mL vs. 5.3 (4.5–7.1) pg/mL, *P* < 0.001} (Figure 1). According to the ROC curve analysis, the optimal cut-off value for the serum NFL levels to discriminate ALS from the HCs was 14.3 pg/mL with a sensitivity of 100% and a specificity of 100%.

### The Clinical Relevance of Serum NFL in the ALS Patients

**Figure 2:**
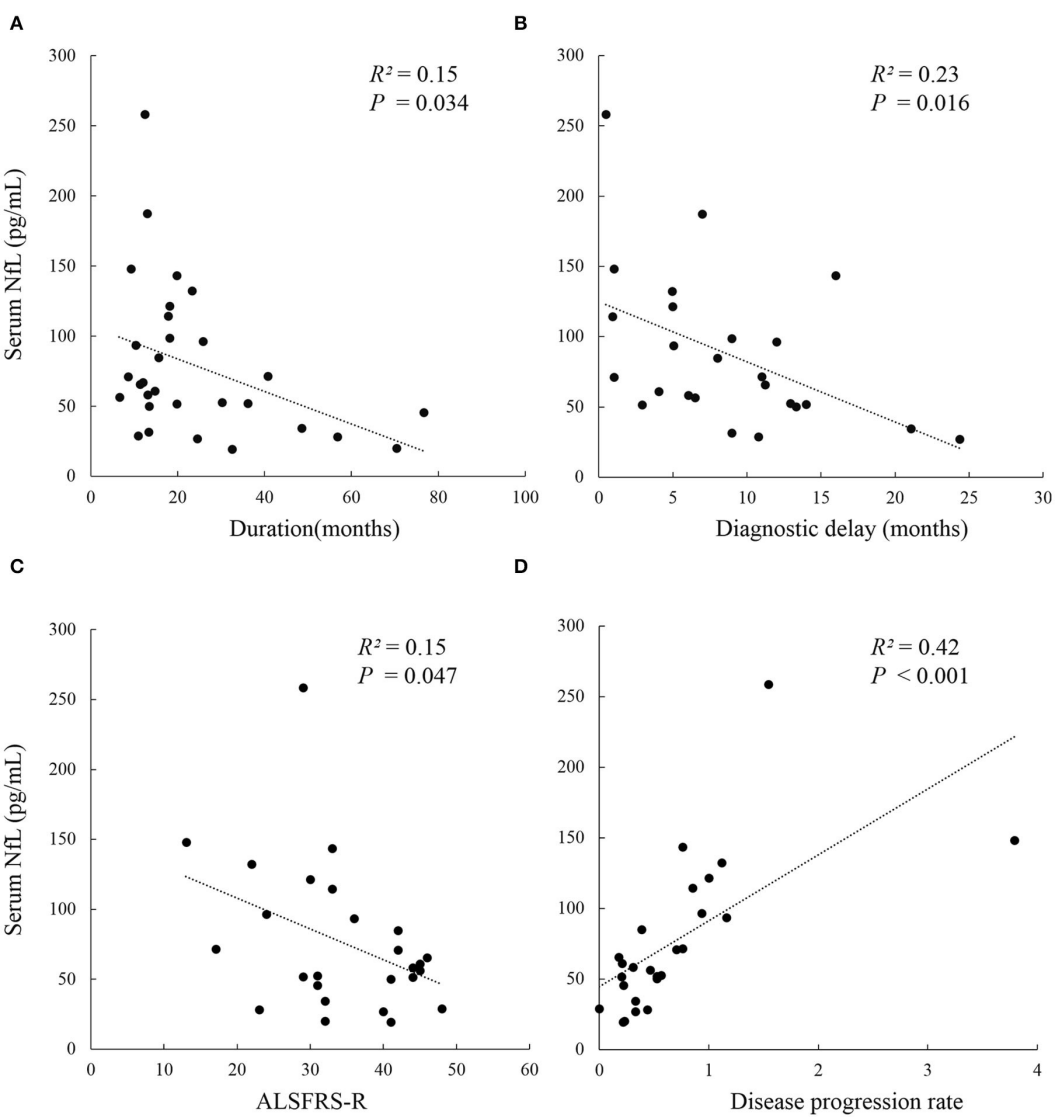
Correlation analysis between serum NFL level and clinical parameters in Chinese patients with ALS. **(A)** Serum NFL level negatively correlated with disease duration (*R*^2^ = 0.15, *P* = 0.034); **(B)** Serum NFL level negatively correlated with diagnostic delay (*R*^2^ = 0.23, *P* = 0.016); **(C)** Serum NFL level negatively correlated with ALSFRS-r (*R*^2^ = 0.15, *P* = 0.047); **(D)** Serum NFL level positively correlated with the disease progression rate (*R*^2^ = 0.42, *P* < 0.001). ALS, Amyotrophic lateral sclerosis; ALSFRS-r, Amyotrophic lateral sclerosis functional rating scale revised; NFL, Neurofilament light chain.

The serum NFL levels did not differ across onset sites (bulbar or spinal onset) (*P* = 0.447), but were significantly higher in rapidly progressive ALS than in slowly progressive ALS (*P*_DPR_ < 0.001, *P*_Diagnosticdelay_ = 0.019). In addition, patients in disease Stage 2 had lower levels of serum NFL than those in Stage 3 [median (IQR): 54.1 (36.3–60.3) pg/mL vs. 98.6 (68.5–132.5) pg/mL, *P*_*stage*2*vs*.3_ = 0.033], but they showed no significant difference in serum NFL levels compared to those in Stage 4. Moreover, the serum NFL levels negatively correlated with the diagnostic delay (*R*^2^ = 0.23, *P* = 0.016), ALSFRS-r score (*R*^2^ = 0.15, *P* = 0.047), and disease duration (*R*^2^ = 0.15, *P* = 0.034), and positively correlated with the DPR (*R*^2^ = 0.42, *P* < 0.001), but did not correlate with the disease stage (*P* = 0.320). In addition, the disease stage positively correlated with the disease duration (*r* = 0.476, *P* = 0.008) and the DPR (*r* = 0.446, *P* = 0.022) and negatively correlated with the ALSFRS-r score (*r* = −0.810, *P* < 0.001) in the ALS group (Figure 2, Tables 2, 3).

**Table 2:** Serum NFL levels in the different subgroups of patients with ALS.

**Variable**	Serum NFL (pg/mL)^*^	***P*-value**
**Site of onset**		
Limb, *n* = 22	59.7 (38.9–91.4)	*P* = 0.447^**§**^
Bulbar, *n* = 8	84.0 (49.1–119.0)	
**Diagnostic delay (months)**		
≤ 8.0, *n* = 13	93.5 (61.0–132.4)	***P*** **= 0.019**^**§**^
> 8.0, *n* = 12	52.3 (33.9–77.8)	
**DPR**		
≤ 0.49/month, *n* = 13	45.8 (28.3–58.3)	***P*** **< 0.001**^**§**^
> 0.49/month, *n* = 13	96.4 (71.1–132.4)	
**Clinical stage**		
Stage 2, *n* = 10	54.1 (36.3–60.3)	***P**_***stage*2**_*_**vs.3**_ **= 0.033**^**‡**^
Stage 3, *n* = 11	98.6 (68.5–132.5)	*P_*stage*3_*_vs.4_ = 0.358^**‡**^
Stage 4, *n* = 9	67.0 (28.3–96.4)	*P_*stage*2_*_vs.4_ = 0.718^**‡**^

*ALS, amyotrophic lateral sclerosis; ALSFRS-r, amyotrophic lateral sclerosis functional rating scale revised; DPR, Disease progression rate; NFL, Neurofilament light chain*.

* *Data are presented as median (interquartile range)*.

§ *The Mann–Whitney U-test*.

‡ *The Steel-Dwass multiple comparison*.

*Bold type indicates a significant difference*.

**Table 3:** The correlation between the disease stage and clinical parameters of patients with ALS.

	***n***	**Spearman *r***	***P*-value**
**Clinical Disease Stage (2, 3, 4)**			
Serum NFL level	30	0.188	*P* = 0.320
Disease duration (months)	30	0.476	***P*** **= 0.008**
ALSFRS-r	26	−0.810	***P*** **< 0.001**
DPR	26	0.446	***P*** **= 0.022**

*ALS, amyotrophic lateral sclerosis; ALSFRS-r, amyotrophic lateral sclerosis functional rating scale revised; DPR, Disease progression rate; NFL, Neurofilament light chain*.

*Bold type indicates a significant difference*.

## Discussion

The present study preliminarily investigated the serum NFL level and its clinical relevance, including its association with disease severity and progression, in patients with ALS in China. The results demonstrated significantly more elevated levels of serum NFL in patients with ALS than in the HCs, and the serum NFL levels were significantly higher in rapidly progressive ALS and patients in Stage 3 than in slowly progressive ALS and patients in Stage 2. Additionally, the serum NFL levels negatively correlated with the diagnostic delay, the ALSFRS-r score, and disease duration, and positively correlated with the DPR.

NFL, the primary component of the neuronal cytoskeleton, is released to the CSF when axonal and motor neuron impairment occurs (14). Due to the existence of the blood-brain barrier, a higher concentration of NFL is found in the CSF compared to its concentration in the blood (17). In the past, due to the lower sensitivity of the traditional detection method, NFL levels in the CSF have been used as the best indicator to identify ALS and for patient stratification. With the availability and application of Simoa technology, the NFL levels in both the CSF and serum can be detected with high sensitivity and accuracy (15, 16). Moreover, compared to an invasive lumbar puncture for CSF collection, serological specimens are more convenient and easily accessible, and a number of studies have proven that CSF NFL levels positively correlate with the serum NFL level (14, 17). This has made serum NFL a valid substitute for CSF NFL to conduct a series of studies in ALS (14). In this study, the NFL level was detected using ultrasensitive Simoa technology in the serum, and this study showed elevated serum NFL levels in patients with ALS in China, which is consistent with the results of previous studies in Western countries. This result further proves the potential diagnostic value of serum NFL for the Chinese ALS population (11, 14, 18, 25–27). However, to evaluate the diagnostic performance and further establish a more accurate cut-off value of the serum NFL level for the diagnosis of ALS, the serum levels of NFL need to be investigated in a larger number of Chinese ALS patients and disease controls, including patients with other neurodegenerative diseases and non-neurodegenerative diseases.

As for the clinical relevance of serum NFL, it was found that the serum NFL level was significantly higher in rapidly progressive ALS than that in slowly progressive ALS. Moreover, the serum NFL level positively correlated with the DPR and negatively correlated with the diagnostic delay, two indicators of disease progression in ALS. It is believed that several reasons could account for the diagnostic delays of ALS patients, such as how soon patients seek medical help after disease onset, their social-economic situations, their accessibility to a tertiary neurologic clinic, and other factors. The disease progression indicated the clinical deterioration, which was largely due to the biological aggressiveness of the disease, and as described before, one of the primary sources of serum NFL is release from impaired axons and neurons. Higher serum levels of NFL were associated with a rapid disease progression in patients with ALS, as described above, and this may also indicate the intensity of the neurodegenerative process and may point toward a rapid progression of the disease in its early phase. The results above were consistent with previous reports of other ethnicities, and this indicated serum NFL could be viewed as a reliable biomarker to predict disease progression in the Chinese ALS population (11, 12, 14, 17, 28). ALSFRS-r is a cumulative score that evaluates the neurological functional disability, while the staging system describes the anatomical or pathological spread of a disease, both of which are indicators of disease severity. In this study, the disease stage negatively correlated with the ALSFRS-r score. This may suggest the pathological or anatomical damage in the CNS that eventually leads to neurological disability. And the severity of the neurological disability could in turn indicate the anatomical extent of the injury indirectly, and as reported previously, the disease stage can be estimated using the ALSFRS-r score with 92% concordance (9). Moreover, the present study revealed that the serum NFL levels negatively correlated with the ALSFRS-r score, which indicated that the serum NFL level could reflect the degree of neurological disability. However, a conclusion regarding whether serum NFL levels could reflect the anatomical extent of the CNS in ALS could not be determined. This is because patients in Stage 2 had lower levels of serum NFL than those in Stage 3, but did not significantly differ from those in Stage 4. Additionally, the serum NFL level had no correlation with the disease stage in ALS. The above results may have been partially due to the limited number of ALS patients in each disease stage. In summary, the present study showed that serum NFL levels were associated with disease severity and progression, but due to the limited number of enrolled patients with ALS, the conclusion should be considered with caution. In addition, this study showed a negative correlation between the serum NFL level and disease duration, which was consistent with some other reports (11, 14), but was in disagreement with others that indicated a flat serum NFL concentration profile after a robust increase in the early stage of the disease (17). Therefore, a longitudinal study that uses a series of serum NFL detections with a large number of patients will be needed to clarify the fluctuations in the serum NFL levels throughout the entire disease course and its association with the clinical progression of ALS.

The present study had some limitations. First, due to the rarity of ALS, the number of patients included in this study was limited, which made the availability of subgroup analysis limited. Second, this study lacked disease controls, which made a detailed evaluation of the diagnostic performance of serum NFL impossible. However, this study had preliminarily explored the feasibility of applying the ultrasensitive Simoa technology to detect serum NFL levels in the healthy population and ALS patients in China, which laid a foundation for further research. Third, patient clinical data was collected in a retrospective manner, and there was no prospective longitudinal follow-up detection of the level of serum NFL, which made clarification of changes in the serum NFL level with the disease progression impossible. Fourth, we lacked the evaluation of the association between the serum levels of NFL and the extent of upper and lower motor neuron disease. Therefore, the conclusions of the present study are very preliminary and should be considered with caution. Further prospective longitudinal studies with a greater number of patients and appropriate disease controls will be needed to confirm the present study's findings and further evaluate the diagnostic performance and clinical relevance of serum NFL in Chinese patients with ALS.

## Conclusions

The present study preliminarily investigated the diagnostic value of serum NFL and its clinical relevance in the Chinese ALS population using the ultrasensitive Simoa technology. The results demonstrated that the level of serum NFL may become a potential biomarker for ALS diagnosis and indicate disease severity and progression.

## Data Availability Statement

The raw data supporting the conclusions of this article will be made available by the authors, without undue reservation.

## Ethics Statement

The studies involving human participants were reviewed and approved by Ethics Committee of Dongzhimen Hospital Affiliated to Beijing University of Chinese Medicine. The patients/participants provided their written informed consent to participate in this study.

## Author Contributions

KS contributed to conception and design, acquisition, analysis and interpretation of data, statistical analysis, and drafting and revision of the manuscript. YH and YS contributed to the acquisition of data and revision of the manuscript. YG was the study supervisor. All authors contributed to the article and approved the submitted version.

## Conflict of Interest

The authors declare that the research was conducted in the absence of any commercial or financial relationships that could be construed as a potential conflict of interest.
